# Impacts of meteorological factors on the risk of scrub typhus in China, from 2006 to 2020: A multicenter retrospective study

**DOI:** 10.3389/fmicb.2023.1118001

**Published:** 2023-02-23

**Authors:** Ling Han, Zhaobin Sun, Ziming Li, Yunfei Zhang, Shilu Tong, Tian Qin

**Affiliations:** ^1^State Key Laboratory for Infectious Disease Prevention and Control, National Institute for Communicable Disease Control and Prevention, Chinese Center for Disease Control and Prevention, Beijing, China; ^2^Institute of Urban Meteorology, China Meteorological Administration, Beijing, China; ^3^China Meteorological Administration Urban Meteorology Key Laboratory, Beijing, China; ^4^Shanghai Children’s Medical Center, Shanghai Jiao Tong University School of Medicine, Shanghai, China; ^5^School of Public Health, Institute of Environment and Population Health, Anhui Medical University, Hefei, China; ^6^Center for Global Health, Nanjing Medical University, Nanjing, China; ^7^School of Public Health and Social Work, Queensland University of Technology, Brisbane, QLD, Australia

**Keywords:** scrub typhus, rickettsiosis, vector-borne diseases, climatic factors, risk window, regional heterogeneity

## Abstract

Scrub typhus is emerging as a global public health threat owing to its increased prevalence and remarkable geographic expansion. However, it remains a neglected disease, and possible influences of meteorological factors on its risk are poorly understood. We conducted the largest-scale research to assess the impact of meteorological factors on scrub typhus in China. Weekly data on scrub typhus cases and meteorological factors were collected across 59 prefecture-level administrative regions from 2006 to 2020. First, we divided these regions into 3 regions and analyzed the epidemiological characteristics of scrub typhus. We then applied the distributed lag nonlinear model, combined with multivariate meta-analysis, to examine the associations between meteorological factors and scrub typhus incidence at the total and regional levels. Subsequently, we identified the critical meteorological predictors of scrub typhus incidence and extracted climate risk windows. We observed distinct epidemiological characteristics across regions, featuring obvious clustering in the East and Southwest with more even distribution and longer epidemic duration in the South. The mean temperature and relative humidity had profound effects on scrub typhus with initial-elevated-descendent patterns. Weather conditions of weekly mean temperatures of 25–33°C and weekly relative humidity of 60–95% were risk windows for scrub typhus. Additionally, the heavy rainfall was associated with sharp increase in scrub typhus incidence. We identified specific climatic signals to detect the epidemic of scrub typhus, which were easily monitored to generalize. Regional heterogeneity should be considered for targeted monitoring and disease control strategies.

## 1. Introduction

Climate can affect the occurrence and transmission of infectious diseases through various avenues, such as the proliferation rate of pathogens, the growth and propagation rate of vectors, and the social behavior of people (Semenza et al., 2012; Franklinos et al., 2019). In recent decades, the emergence of infectious disease has exhibited unprecedented changes, such as variations in the seasonal patterns and altitudinal distribution, the northward expansion and the dispersion into non-endemic localities (Medlock and Leach, 2015). Although the reasons behind this phenomenon are complex, it is certain that climate change is one of the important drivers (Rocklöv and Dubrow, 2020). Therefore, exploring the impacts of meteorological factors on infectious diseases are vitally important for elucidating their transmission mechanisms and establishing early warning systems.

Scrub typhus is an acute febrile zoonosis caused by infection with *Orientia tsutsugamushi* (Walker, 2016). After infection with *O. tsutsugamushi* through the bite of infected larval mites, a typical eschar may appear at the site of biting, followed by chills or high fever, headache, myalgia, lymphadenopathy, and gastrointestinal symptoms. In severe infections, the illness can progress to multiple organ dysfunction syndrome to death (Kelly et al., 2002). Scrub typhus is widely prevalent in a confined geographical realm described as the Asia-Pacific Tsutsugamushi Triangle, which encompasses Pakistan and Afghanistan in the west, far-eastern Russia in the north, Japan in the east, and northern Australia in the south. In recent years, the prevalence of scrub typhus has been increasing, and it has been expanding geographically, even outside of the tsutsugamushi triangle (Xu et al., 2017). In China, scrub typhus was reincorporated as a reportable disease in 2006 due to its remarkably increasing incidence in recent decades. China has also witnessed an expansion of the geographic distribution of scrub typhus from south to north (Yao et al., 2019). It was estimated that more than 192 (95% CI: 187–195) million people lived in areas of potential scrub typhus infection in China (Zheng et al., 2019).

Even with its escalating threat, scrub typhus remains a neglected vector-borne disease, and the evidence regarding its prevalence and transmission mechanisms is limited (Walker, 2016). Although several studies have investigated the impact of meteorological factors on the epidemiology of scrub typhus, the results remain inconsistent (Li et al., 2014; Seto et al., 2017; Wei et al., 2017; Lu et al., 2021). For example, due to differences in the selected meteorological factors, study locations and methods, the reported meteorological factors which were critical to the incidence of scrub typhus were inconsistent in previous studies, and the quantitative results of meteorological factors on the scrub typhus risk were also discrepant. For most of the studies, the location of research in a single area renders the results difficult to generalize, and the different statistical methods make the results incomparable. Additionally, restricted by the accessibility of data, previous studies are mostly conducted at yearly or monthly scale, which is unfavorable to provide more timely information for the prevention and control of scrub typhus according to changes in weather conditions. As a result of continuous warming of the climate, the patterns of temperature and precipitation will experience tremendous variation, driving changes in the environmentally suitable conditions of infectious disease (Liang and Gong, 2017; Rocklöv and Dubrow, 2020). The rapid process of urbanization in China will also cause dramatic changes in natural landscapes and ecological fragmentation, altering the meteorological potential for the transmission of scrub typhus (Gong et al., 2012; Baker et al., 2022). Meanwhile, China is a big agricultural country and a large population labors in the field. These factors will pose direct and indirect threats to the occurrence of scrub typhus, and ultimately multiply the existing problem of the disease.

Therefore, we conducted a multicenter study in China with the following aims: (a) to investigate the epidemiology of scrub typhus; (b) to assess the impacts of different meteorological factors on scrub typhus incidence; (c) to estimate the regional heterogeneity of those impacts; and (d) to identify climate risk windows for scrub typhus.

## 2. Materials and methods

### 2.1. Scrub typhus cases

**Figure 1:**
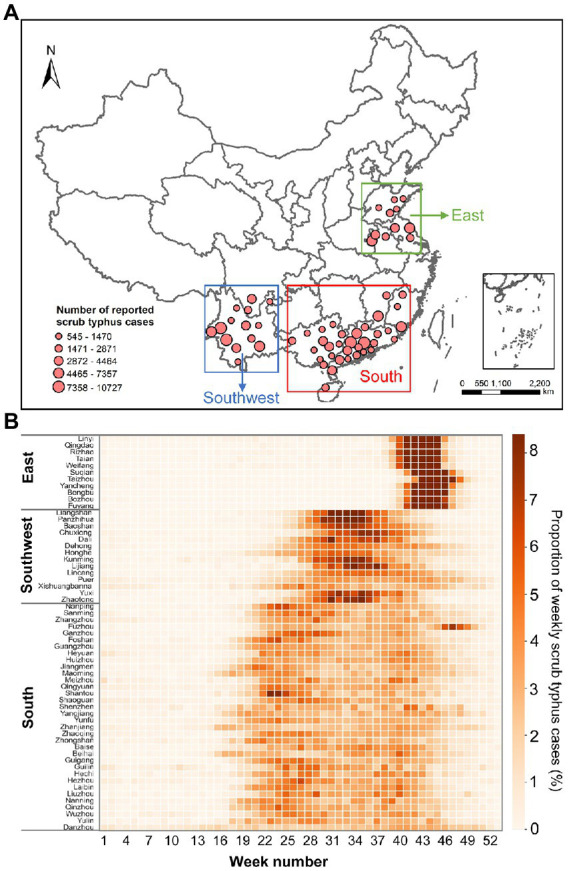
**(A)** Geographic locations of the 59 prefecture-level administrative regions included in the study and the total reported scrub typhus cases during 2006–2020. **(B)** Seasonal characteristics of scrub typhus cases. Each cell denotes the proportion of weekly scrub typhus cases out of the total scrub typhus cases.

Since 2006, all scrub typhus cases have been reported *via* the National Notifiable Infectious Disease Reporting Information System (NNIDRIS) of the China Center for Disease Control and Prevention (China CDC). This time-series analysis included weekly typhus cases from 59 prefecture-level administrative regions in 10 provinces in mainland China from January 1, 2006, to December 31, 2020 (Figure 1). This study included all clinically diagnosed cases and laboratory-confirmed cases.

The diagnostic criteria for suspected scrub typhus cases (meet criteria 1 and 2.1, and meet 1 of criteria 2.2 and 2.3), clinical scrub typhus cases (meet the diagnostic criterion of suspected scrub typhus and meet criterion 2.4; or meet criteria 1, 2.1, and 2.4), and confirmed scrub typhus cases (meet the diagnostic criterion of suspected scrub typhus and meet 1 of the criteria of 3.2, 3.3, and 3.4; meet the diagnostic criterion of clinical scrub typhus and meet 1 of criteria 3) were identified according to the guidebook for prevention and control of scrub typhus issued by the China CDC (Chinese Center for Disease Control and Prevention, 2009). One field exposure history: A person who had a field exposure history of being in a scrub typhus-endemic area 3 weeks before the onset of illness during the scrub typhus transmission season: 2 Clinical manifestations. 2.1 A person who had clinical manifestations of high fever; 2.2 A person who had clinical manifestations of lymphadenopathy; 2.3 A person who had clinical manifestations of skin rash; 2.4 A person who had clinical manifestations of typical cutaneous lesions (eschars or ulcers); 3 Laboratory tests: 3.1 Weil-Felix OX-K agglutination titer ≥ 1:160; 3.2 A 4-fold or greater rise in serum IgG antibody titers between acute and convalescent sera as detected by indirect immune fluorescence antibody assay (IFA); 3.3 Positive results of *O. tsutsugamushi* in clinical specimens by polymerase chain reaction (PCR); 3.4 Isolation of *O. tsutsugamushi* from clinical specimens.

### 2.2. Meteorological data

Meteorological data from January 1, 2006, to December 31, 2020, including mean temperature, relative humidity, air pressure, mean wind velocity, cumulative rainfall, and cumulative sunshine hours, were derived from the China Meteorological Data Sharing Service System. We chose the National Basic Weather Station (NBWS) for each administrative region because of the better quality of meteorological data. For prefecture-level administrative regions with more than 1 NBWS, the data for these NBWS units was averaged to represent the meteorological conditions of the region. We initially obtained meteorological data at the daily level and then aggregated them on a weekly scale. Data on weekly mean temperature, relative humidity, air pressure, and wind velocity were the mean values of the corresponding week, while weekly cumulative rainfall and cumulative sunshine hours were the sum values of the corresponding week.

### 2.3. Statistical analysis

The associations between meteorological factors and scrub typhus incidence were reported as relative risk (RR). To determine the reference value of each meteorological factor, we generated heat maps between each meteorological factor interval and its corresponding weekly mean scrub typhus cases, and the results indicated that there might be no scrub typhus cases under certain weather conditions, such as low temperature and low relative humidity. This means that all 59 prefecture-level administrative regions reported scrub typhus cases when the value of the meteorological factors exceeded a certain threshold, and such thresholds were set as references.

We utilized a two-stage analysis to establish the exposure-response associations between meteorological elements (Gasparrini et al., 2015). In the first stage, we applied a standard time-series quasi-Poisson regression with distributed lag nonlinear model (DLNM) to derive the associations between meteorological factors and scrub typhus incidence in each prefecture-level administrative region. The model is described as follows.


$$\begin{array}{l} \log\left[E\left(Y_{t}\right)\right]=\alpha +cb\left(\text{meteorological variable},\text{lag},df\right)+\\ \;\;\;\;\;\;\;\;\;\;\;\;\;\;\;\;\;\;\;\;\;\;\;\;\;\sum ns\left(X_{j},df\right)+ns\left(\text{time},df\right) \end{array}$$

where *E(Y_t_)* denotes the weekly scrub typhus cases in week *t*, *α* is the intercept, *cb()* denotes the cross-basic function modeling the nonlinear and lagged impacts of meteorological factors on scrub typhus, *ns()* denotes the cubic spline function, and *time* denotes the calendar weeks to control for the long-term and seasonal trends of scrub typhus using natural cubic splines of time with 7 degrees of freedom (*df*) according to the Akaike’s information criterion (AIC). *Xj* denotes the meteorological variables other than the factor in the cross-basic matrix. For example, when the impact of mean temperature on scrub typhus incidence was evaluated, the potential impacts of other factors were controlled by natural cubic splines with 3 degrees of freedom. Experimental ecology studies have shown that it may take more than 2 months for the eggs of chigger mites to develop into larvae (Li et al., 2002), and the incubation period of scrub typhus is generally 4–21 days. Previous studies found that the impact of meteorological factors on scrub typhus incidence can be delayed to more than 3 months (Yang et al., 2014; Lu et al., 2021). Therefore, in this study, a maximum of 15 weeks was used to capture the lagged effects of meteorological factors on scrub typhus.

In the second stage, a multivariate meta-analysis was performed to obtain the overall associations between meteorological factors and scrub typhus at both the total and regional levels.

Our initial analysis indicated a strong negative correlation between weekly mean temperature and air pressure. To avoid collinearity, air pressure was not included in the final analysis. We separately pooled the exposure-response associations of meteorological factors with scrub typhus incidence. The results indicated that the mean temperature and relative humidity were 2 critical climatic factors for scrub typhus incidence. To further explore the comprehensive impacts of mean temperature and relative humidity on scrub typhus, i.e., extracting the climate risk windows, we separately calculated the average values of the Spearman correlation coefficients of mean temperature and relative humidity with scrub typhus cases over 0–15 lag weeks in each administrative region. We then generated heat maps of scrub typhus cases against mean temperature and relative humidity in the lag week with the strongest correlations and identified the climate risk window for scrub typhus in each administrative region, which was defined as the range of mean temperature and relative humidity within which the number of scrub typhus cases exceeded the 95th percentile of the entire distribution of scrub typhus cases in the corresponding administrative region. Furthermore, we gathered the climate risk window for each administrative region and extracted a common risk window for scrub typhus.

In addition, we conducted sensitivity analyzes by altering the maximum lag time and the *df* value of time. Sensitivity analyzes were performed for associations of mean temperature and relative humidity with scrub typhus incidence. The above analyzes were conducted using the dlnm and mvmeta packages in the R software (Version R 4.1.1).

## 3. Results

### 3.1. Epidemiological characteristics of scrub typhus

Between 2006 and 2020, 170,582 scrub typhus cases were reported in the 59 prefecture-level administrative regions, which accounted for 87% of the total scrub typhus cases in mainland China. The occurrence of the disease varied greatly across regions, ranging from 545 cases in Shenzhen to 10,727 cases in Baoshan. Among the total cases, only 3% were confirmed by laboratory diagnosis.

Based on the different epidemic characteristics of scrub typhus, we divided the 59 administrative regions into 3 regions; east, southwest, and south (Figure 1). Scrub typhus cases in the 11 prefecture-level administrative regions of Shandong, Jiangsu, and Anhui provinces (i.e., Eastern Region) exhibited distinct clustering features, with short epidemic duration peaks in the 39–46th weeks, equivalent to the period between September and October. Scrub typhus in the 14 administrative regions of Sichuan and Yunnan provinces (i.e., southwest region) also presented obvious clustering features, with the epidemic concentrating in the 30–40th weeks, which correspond to July and August. In contrast, the epidemiological characteristics of scrub typhus in the remaining 34 administrative regions (i.e., southern region) were manifested by more evenly distributed and longer prevalence periods across summer and autumn. The median age of cases in the East and South was 59 and 55, respectively, with the cases aged 40–79 accounting for large proportion. However, the median age of cases in the Southwest was 42, with the larger proportion of cases younger than 9 years in the Southwest than in the East and South (Supplementary Table S1; Supplementary Figure S1).

### 3.2. Impacts of meteorological factors on the risk of scrub typhus

#### 3.2.1. Reference values for different meteorological factors

**Figure 2:**
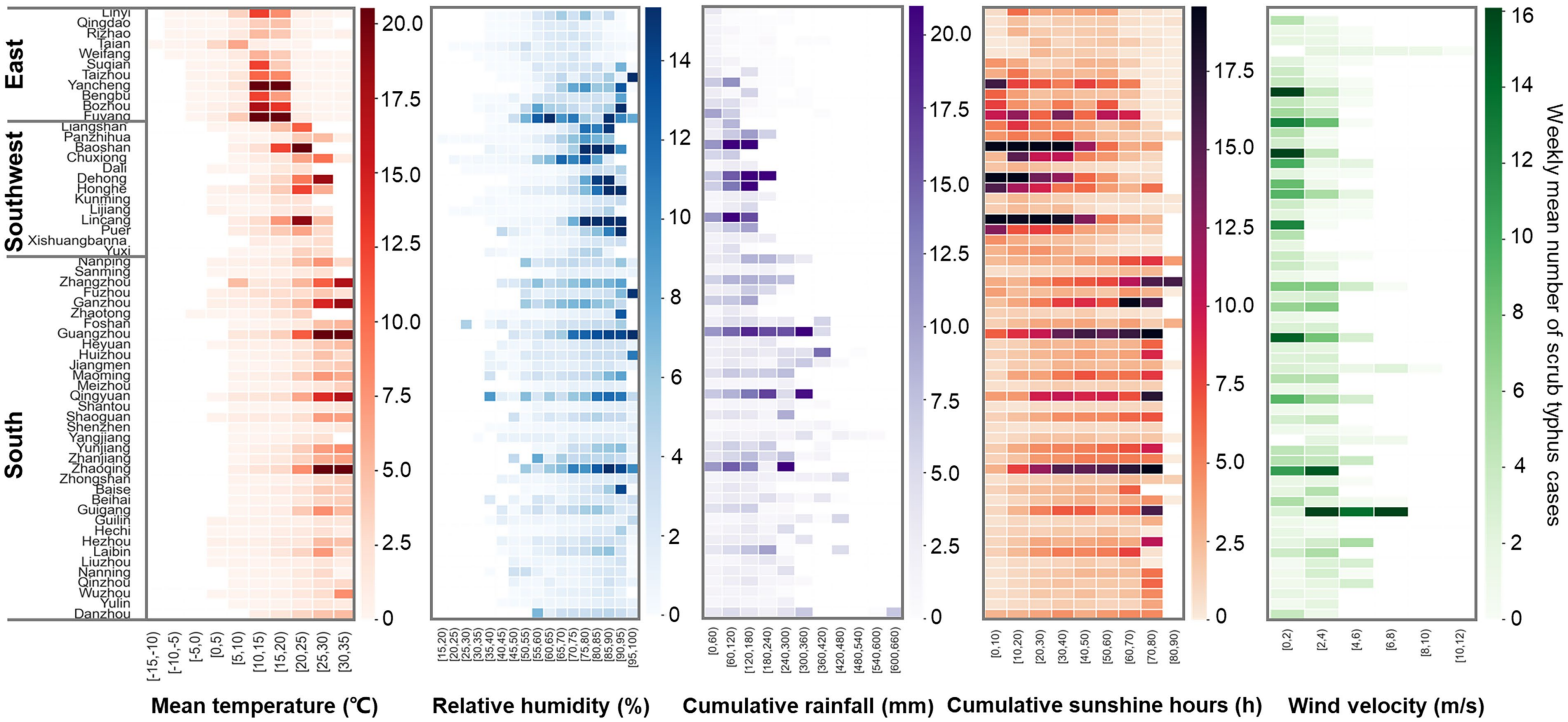
Heatmaps of scrub typhus cases against meteorological factors. Each cells denotes the weekly mean scrub typhus cases under the corresponding climate conditions.

Heat maps of meteorological factors against scrub typhus cases showed that scrub typhus cases generally increased with increments in weekly mean temperature and relative humidity (Figure 2). Specifically, when the weekly mean temperature was ≥ 10°C and weekly relative humidity was ≥ 60%, all administrative regions reported scrub typhus cases. Therefore, a mean temperature of 10°C and relative humidity of 60% were set as the reference values to estimate their impact on scrub typhus incidence. However, there were no threshold for the associations of scrub typhus cases with other factors, thus, reference values for cumulative rainfall, cumulative sunshine hours and mean wind velocity were set as zero.

#### 3.2.2. Exposure-response associations between meteorological factors and scrub typhus incidence

**Figure 3:**
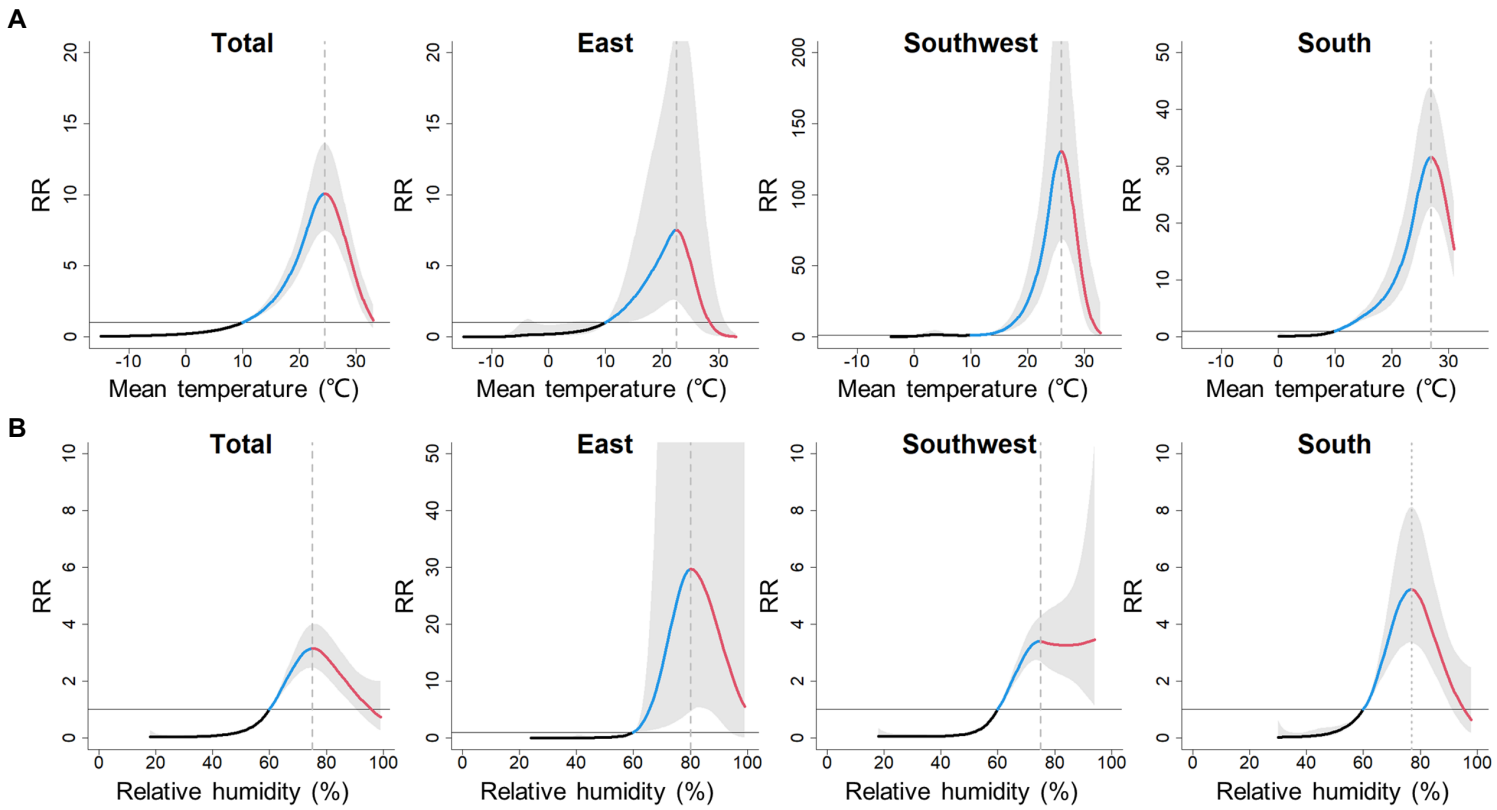
Pooled cumulative exposure-response curves for associations between weekly **(A)** mean temperature and **(B)** relative humidity, with scrub typhus incidence over 0–15 lag weeks at total and regional levels. Black, blue and red solid lines denote the meta-pooled estimates, indicating the initinal phase, elevated phase and descendent phase, respectively; shaded areas denote the 95% confidence intervals; the reference value for mean temperature and ralative humidity are 10°C and 60%, respectively.

As the temperature increased, the incidence of scrub typhus first remained stable, then gradually increased to its peak and proceeded further into a decrease (Figure 3A). Therefore, temperature-scrub typhus associations could be described as an initial-elevated-descendent pattern. At the total level, the impact of mean temperature on scrub typhus incidence peaked at 24.5°C, with a cumulative RR of 10.10 (95% CI: 7.45–13.60). At the regional level (Table 1), the temperature thresholds were 22.5°C for the East, 25.9°C for the Southwest, and 26.9°C for the South. The cumulative RR was largest for the Southwest.

**Table 1:** Threshold values of weekly mean temperature and relative humidity and the corresponding cumulative relative risk (RR) at total and regional levels.

		Mean temperature	Relative humidity
Total	Threshold	24.5°C	75%
	RR (95% CI)	10.10 (95% CI: 7.45–13.60)	3.15 (95% CI: 2.48–4.01)
East	Threshold	22.5°C	80%
	RR (95% CI)	7.51 (95% CI: 2.54–22.17)	29.73 (95% CI: 4.69–188.48)
Southwest	Threshold	25.9°C	75%
	RR (95% CI)	130.40 (95% CI: 68.4–248.9)	3.40 (95% CI: 2.69–4.30)
South	Threshold	26.9°C	77%
	RR (95% CI)	31.60 (95% CI: 22.8–43.7)	5.23 (95% CI: 3.36–8.14)

Analogously, the associations between relative humidity and scrub typhus incidence also exhibited initial-elevated-descendent phases, except for the Southwest (Figure 3B). At the total level, the impact of relative humidity on scrub typhus incidence peaked at 75%, with a cumulative relative risk of 3.15 (95% CI: 2.48–4.01). At the regional level (Table 1), the relative humidity threshold was 80% for the East and 77% for the South. However, the impact of relative humidity on scrub typhus incidence in the Southwest peaked at 75%, then remained at a high level as the relative humidity continuously rose. The cumulative RR estimates was largest for the East, with a cumulative RR of 29.73 (95% CI: 4.69–188.48).

**Figure 4:**
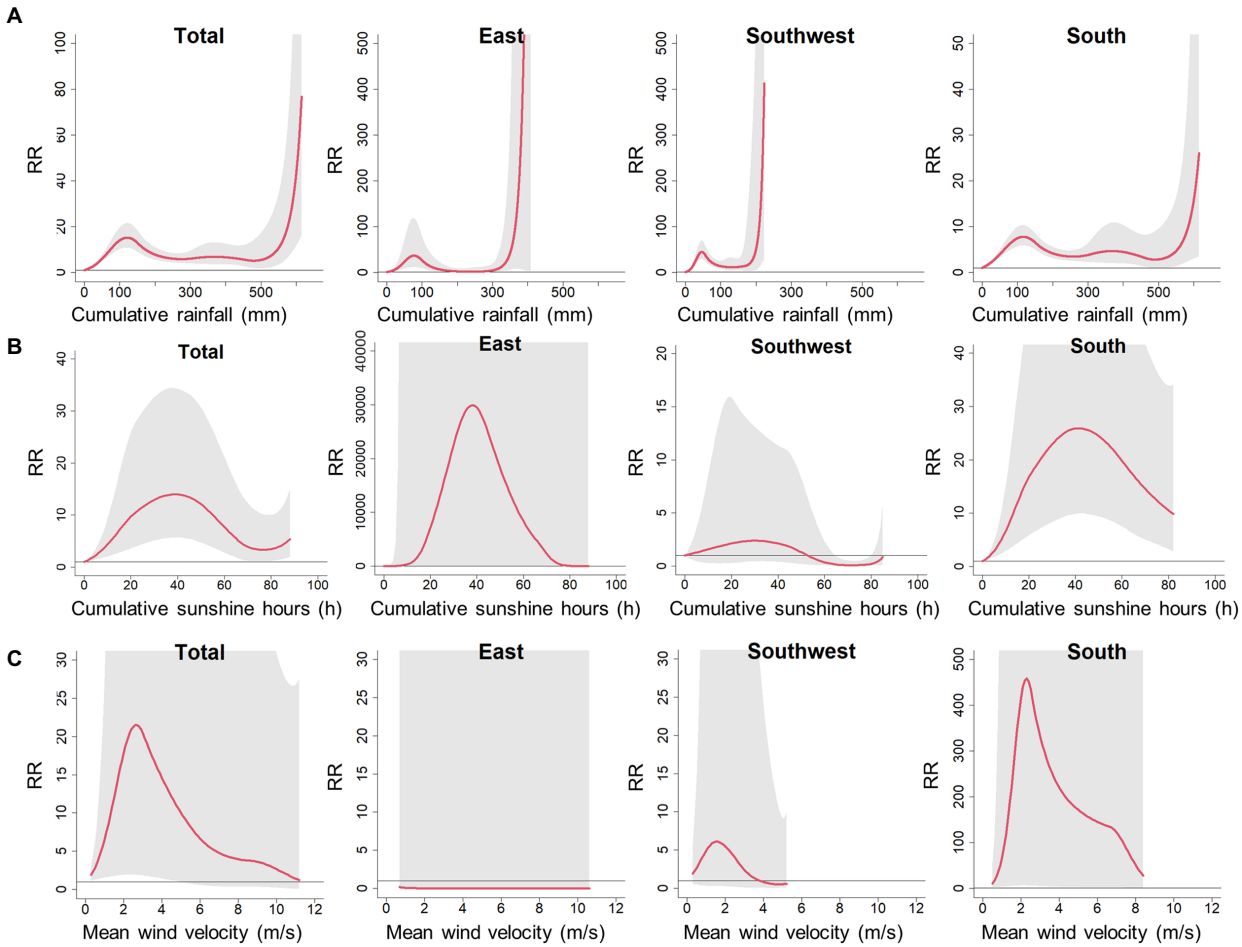
Pooled cumulative exposure-response curves for associations between weekly **(A)** cumulative rainfall, **(B)** cumulative sunshine hours and **(C)** mean wind welocity, with scrub typhus incidence over 0–15 lag weeks at total and regional levels. Red solid lines denote the meta-pooled estimates; shaded areas denotes 95% confidence intervals; the reference value for cumulative rainfall, cumulative sunshine hours and mean wind welocity are 0 mm, 0 h and 0 m/s, respectively.

The association between cumulative rainfall and scrub typhus incidence indicated that heavy rainfall was associated with a sharp increase in scrub typhus risk (Figure 4A). For the impacts of cumulative sunshine hours and mean wind velocity, the results generally exhibited inverse “V” shapes (Figures 4B,C). However, these results on association of cumulative sunshine hours and mean wind velocity with scrub typhus appear to have large uncertainties with wide 95% confidence intervals. Therefore, in the following analysis, we further explored the climate risk windows for scrub typhus, signified by the comprehensive effects of mean temperature and relative humidity. The reason for the cumulative rainfall not included was that cumulative rainfall has a high correlation with relative humidity, with a Spearman correlation coefficient of 0.62 in the East (*p* < 0.001), 0.62 in the Southeast (*p* < 0.001), and 0.61 in the South (*p* < 0.001), respectively. In the sensitivity analyzes, there were no significant changes in the associations of scrub typhus with mean temperature and relative humidity (Supplementary Figures S2–S5), suggesting robustness of the estimates obtained from the core model.

### 3.3. Climate risk windows for scrub typhus incidence

As the lag weeks extended, the Spearman correlation coefficients of weekly scrub typhus cases with mean temperature and relative humidity first gradually increased and then decreased in all administrative regions. However, heterogeneity also existed across regions (Supplementary Table S2). Correlations of mean temperature and relative humidity with scrub typhus were strongest during lag weeks 1–7 in the southwest and south regions, which was earlier than in the east region.

**Figure 5:**
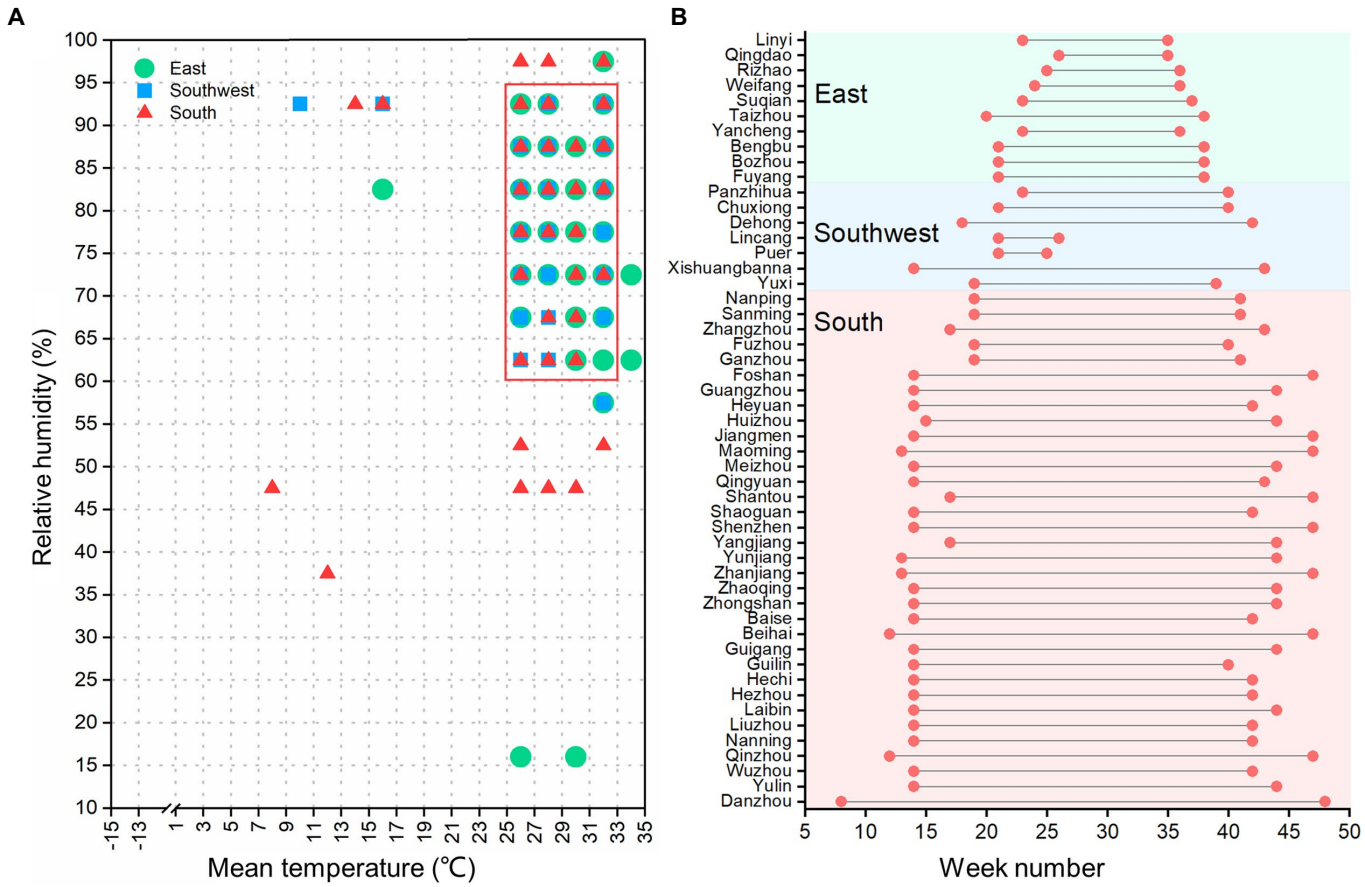
**(A)** Climate risk windows of scrub typhus incidence in each prefecture-level administrative region. The red frame area denotes the common climate risk window for scrub typhus for all study regions. **(B)** Duration of the common climate risk window weeks in each prefecture-level administrative region, with the first green point denoting the starting week and the second green point denoting the ending week, respectively.

Results of the climate risk windows for scrub typhus in each administrative regions indicated that the hot spots of scrub typhus were distributed in local weather conditions of simultaneously high temperature and high relative humidity, with stronger clustering characteristics in the east and southwest regions than in the south region (Supplementary Figures S6–S8). However, weekly mean temperatures in a range of 25–33°C, together with weekly relative humidity of 60–95%, were the common climate risk windows for all study regions (Figure 5A). For the distribution of the common climate risk windows, there were a much longer duration in the South than in the East and Southwest (Figure 5B), indicating that the south of China has more favorable weather conditions for the incidence of scrub typhus.

## 4. Discussion

To the best of our knowledge, this is the largest study to estimate the nonlinear lagged associations between meteorological factors and scrub typhus in China. The results showed that meteorological factors, particularly mean temperature and relative humidity, had profound effects on scrub typhus incidence, and the associations varied with region. Specific thresholds of temperature and relative humidity in different regions can be used to detect the epidemic precursors of scrub typhus and serve as early warning signals for risk mitigation. More importantly, we identified that weekly mean temperature in the range of 25–33°C together with weekly relative humidity in the range of 60–95% was the common climate risk window for scrub typhus, which should be given special attention to prepare for and prevent outbreaks of scrub typhus.

The crucial role of temperature in the incidence of scrub typhus has been well highlighted by previous studies, with a certain temperature range suitable for the transmission of scrub typhus (Yu et al., 2018; Zheng et al., 2019). Our results indicate that the impact of mean temperature on scrub typhus incidence can be described as an initial-elevated-descendent pattern. The underlying mechanism may be related to the influence of meteorological conditions on the ecology of chigger mites as well as contact patterns between humans and chigger mites. As an ectothermic insect, temperature has been verified to be the most critical factor for the ecology of chigger mites, by influencing their life cycle process, oviposition, life span, activity, and behavior (Elliott et al., 2019). Within a suitable temperature range, both the egg-hatching rate and development speed of the chigger mites were consistent with the increase in temperature; however, the egg-hatching rate significantly dropped when temperature exceeded a certain threshold (Lv et al., 2018; Elliott et al., 2019). Moreover, meteorological conditions can affect the incidence of scrub typhus by influencing the frequency of human contact with chigger mites. As the temperature rises, people tend to have more outdoor activities, increasing the chances of contact with chigger mites; however, when the temperature is too high, people’s opportunities to go outside may diminish, and the scrub typhus risks consequently decrease.

In addition to temperature, relative humidity is an important factor that influences the ecology of chigger mites. Increased relative humidity can provide a moist environment that is favorable for the reproduction and growth of chigger mites. Our results indicate that the risk of scrub typhus increases as the relative humidity rises within a suitable range; however, as relative humidity increases to over 75–80%, the incidence risks of scrub typhus attenuate in the eastern and southern regions, which is generally consistent with previous findings (Zheng et al., 2019; Lu et al., 2021). These observations indicate weather conditions that are appropriate for the incidence of scrub typhus and the heterogeneity in different regions.

Furthermore, we found that heavy cumulative rainfall was associated with a sharp increase in the risk of scrub typhus. Previous studies have reported that scrub typhus cases and the prevalence of chiggers were at high levels during the rainy season (Mahajan et al., 2006). The rainfall has been verified as one of the most important meteorological factors influencing the population size of rodents (Li et al., 2021), which are the hosts of scrub typhus. The abundant rainfall is conducive to the growth of vegetation, thus providing a suitable habitation environment for rodents; moreover, the flourishing vegetation can supply rich food resources favorable for the reproduction and survival of rodents. Additionally, the flooding caused by heavy rainfall can enforce rodents from their burrows into the built environment, thus increasing their contact chances with human (Diaz, 2015; Ferro et al., 2020). As a result, the rodent-borne infectious disease outbreaks have been reported to follow heavy rainfall and flooding disasters (Tian et al., 2015). The findings suggest that the impact of rainfall on scrub typhus is worthy of attention for the planning of response measures to control and prevent outbreaks of the disease.

Additionally, we observed different epidemiological characteristics of scrub typhus across different regions, which were manifested by apparent distinctions in seasonal patterns and epidemic durations. The transmission of scrub typhus is mainly reflective of complex interactions between hosts, vectors, humans, and environment. Therefore, the reasons for the different epidemiological patterns could be attributed to the following aspects. First, the vector types and abundance of infected chigger mites contributed to the seasonal patterns of scrub typhus (Kinoshita et al., 2021). In China, the seasonal fluctuations in human scrub typhus were consistent with variations in the locally dominant vector, such as *Leptotrombidium deliense* in the southern natural foci and *Leptotrombidium scutellare* in the northern natural foci, which caused summer-type epidemic and autumn-type epidemic, respectively (Lv et al., 2018). Second, the distribution of meteorological conditions suitable for the incidence of scrub typhus is different among regions. Results of our study indicated that the temporal distribution of climate risk window for scrub typhus varied across regions, with a much longer duration in the South than in other regions, indicating the conducive meteorological conditions for scrub typhus in the south of China. Third, farmers are at the highest risk of scrub typhus because they work the farmlands and are therefore more likely to be exposed to infective chigger mites. In China, 5 climate zones span the country from north to south, leading to cropping systems that exhibit significant differentiation across climate zones. As an index for the cropping system, the Multiple Cropping Index gradually increases from the north to the south in China (Ding et al., 2015). For example, the Huang-Huai-Hai Plain, which belongs to the East region of China, is the concentrated region with a pattern of 2 crops per year; the Southwest region, the mid and lower reaches of the Yangtze River, and the southern region coexist with patterns of 1, 2, and 3 crops per year; and the pattern of 3 crops per year is mainly distributed in the mid and lower reaches of the Yangtze River and the South. Therefore, heterogeneous meteorological conditions lead to distinct cultivation patterns in different regions, which further influence the transmission of scrub typhus by changing the frequency of rodent-chigger mite-human contacts. Additionally, it should be noticed that there was a larger proportion of cases younger than 9 years in the Southwest than in the East and South, which is related to the large number of left-behind children in the Southwest. The lack of education, guardianship and care for left-behind children, coupled with the poor hygiene habits, make the left-behind children more likely to be exposed to high-risk areas of scrub typhus, such as field, greensward and shrub. The results indicated that children in the Southwest were at high risk of scrub typhus. We should strengthen the health education for these people and enhance their sense of protection.

This study has several limitations. First, although the investigation includes the majority of the scrub typhus cases (86%) of China, the findings cannot be interpreted as nationally representative. In recent years, new natural epidemic foci of scrub typhus have been reported in the north, northwest, and northeast of China. Further studies are needed to understand the influence of weather conditions on the geographic expansion of the disease. Second, we collected scrub typhus cases from a passive surveillance system; therefore, underreporting may be inevitable to some extent. Third, important factors, including the prevalence data of rodents and chigger mite were not obtained because of the lack of field surveys. Additionally, vegetation also has important influence on vector and rodent activities. Further studies that include more independent variables are needed to investigate the multiple drivers of scrub typhus.

## 5. Conclusion

In conclusion, our results provided specific indices for the development of targeted and effective monitoring and disease control strategies against scrub typhus in China. This study further promotes the value of meteorological investigation as a means of predicting pandemics and mitigating associated risks of scrub typhus.

## Data availability statement

The original contributions presented in the study are included in the article/Supplementary material, further inquiries can be directed to the corresponding authors.

## Author contributions

LH: conceptualization, methodology, formal analysis, and writing—original draft. ZS: conceptualization, writing—review and editing, and supervision. ZL: data curation. YZ: formal analysis. ST: writing—review and editing and supervision. TQ: conceptualization, writing—review and editing, supervision, and project administration. All authors read and approved the final version of the manuscript.

## Funding

The work was supported by grants from the National Natural Science Foundation of China (grant numbers 81671985 and 42175184), the Science Foundation for the State Key Laboratory for Infectious Disease Prevention and Control from China (grant number 2019SKLID403), the Public Health Service Capability Improvement Project of the National Health Commission of the People’s Republic of China (grant number 2100409034), and the Key innovation team of China Meteorological Administration (grant numbers CMA2022ZD09).

## Conflict of interest

The authors declare that the research was conducted in the absence of any commercial or financial relationships that could be construed as a potential conflict of interest.

## Publisher’s note

All claims expressed in this article are solely those of the authors and do not necessarily represent those of their affiliated organizations, or those of the publisher, the editors and the reviewers. Any product that may be evaluated in this article, or claim that may be made by its manufacturer, is not guaranteed or endorsed by the publisher.
