# Supplementary material for: Impacts of meteorological factors on the risk of scrub typhus in China, from 2006 to 2020: A multicenter retrospective study
Source: Front Microbiol. 2023 Feb 23;14:1118001. doi: 10.3389/fmicb.2023.1118001 (PMC9996048; doi:10.3389/fmicb.2023.1118001)
Supplement: Supplementary file 1 [file Table_1.pdf]

Supplementary Material

Impacts of meteorological factors on the risk of scrub typhus in China, from 2006 to 2020: a multicenter retrospective study

Ling Han, Zhaobin Sun*, Ziming Li, Yunfei Zhang, Shilu Tong, Tian Qin*

*** Correspondence:** Tian Qin: qintian@icdc.cn; Zhaobin Sun: zbsun@ium.cn

1 Supplementary Figures and Tables

1.1 Supplementary Figures

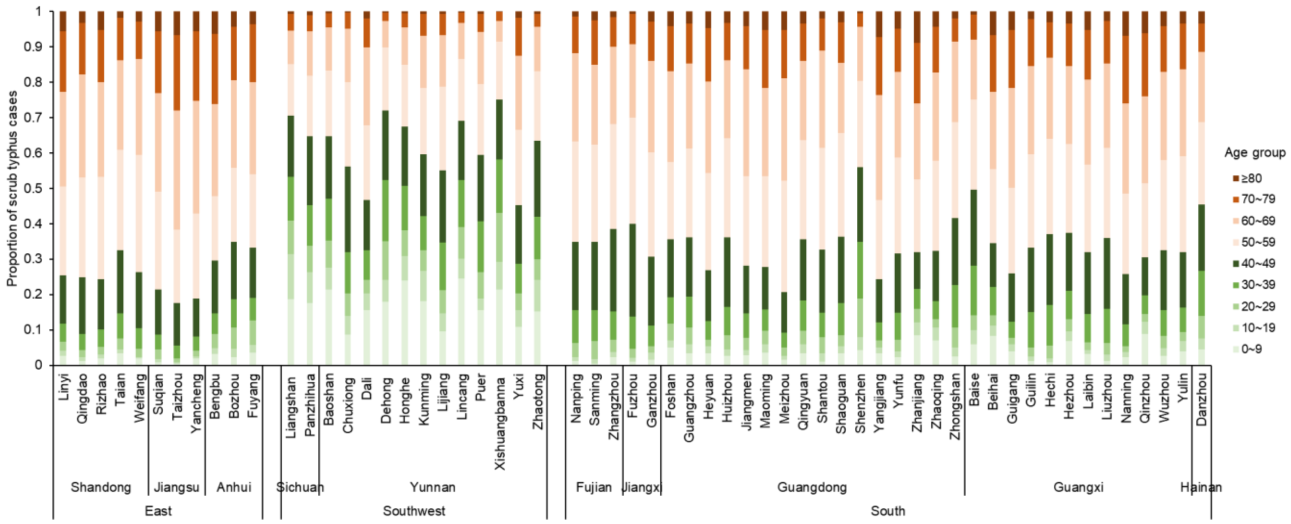

Supplementary Figure 1. Age distribution of scrub typhus cases in the 59 prefecture-level administrative regions during 2006 to 2020.

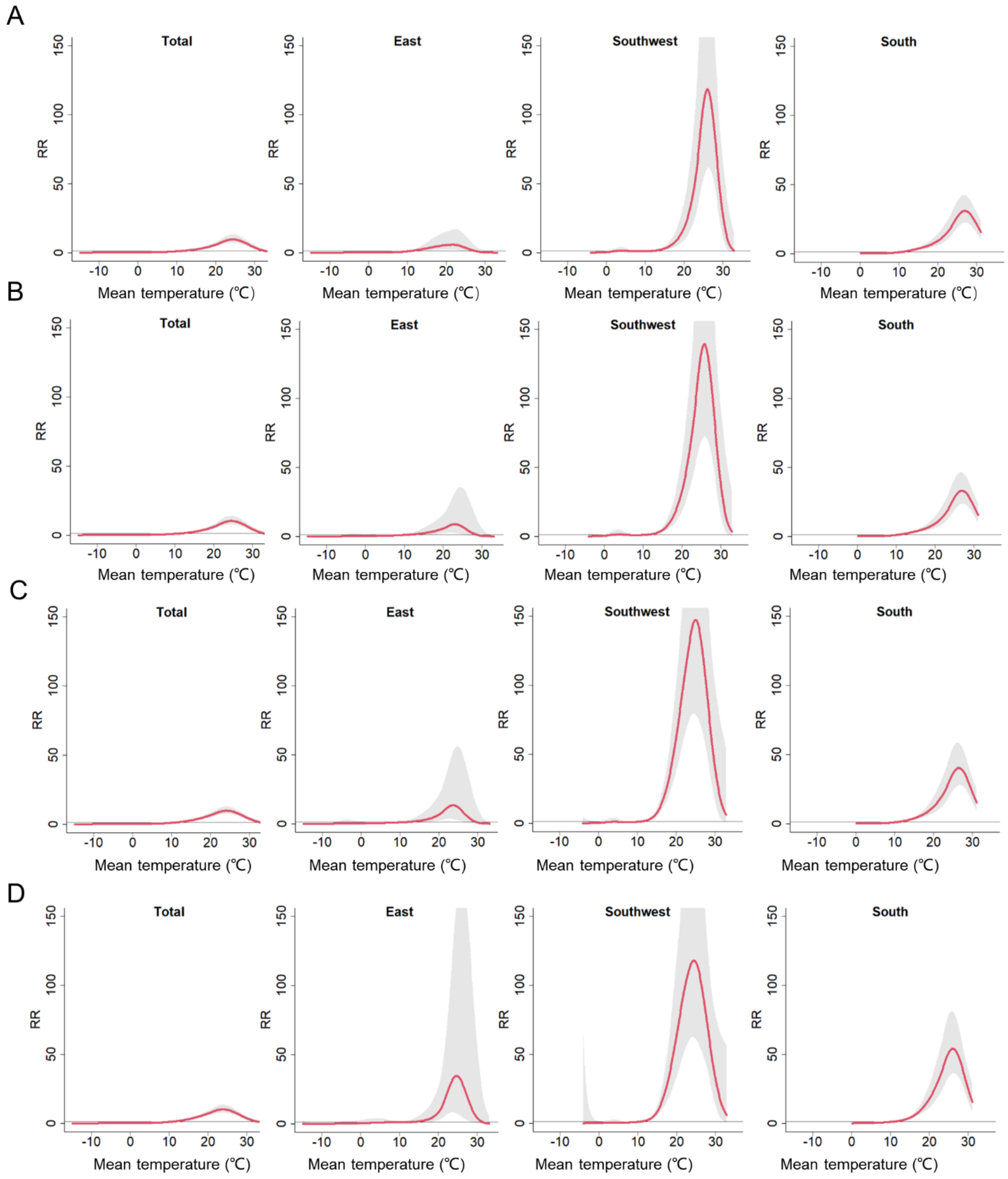

Supplementary Figure 2. Sensitivity analysis of associations between weekly mean temperature and scrub typhus incidence by altering the maximum lag time period to (A) 14, (B) 16, (C) 18 and (D) 20 weeks, respectively.

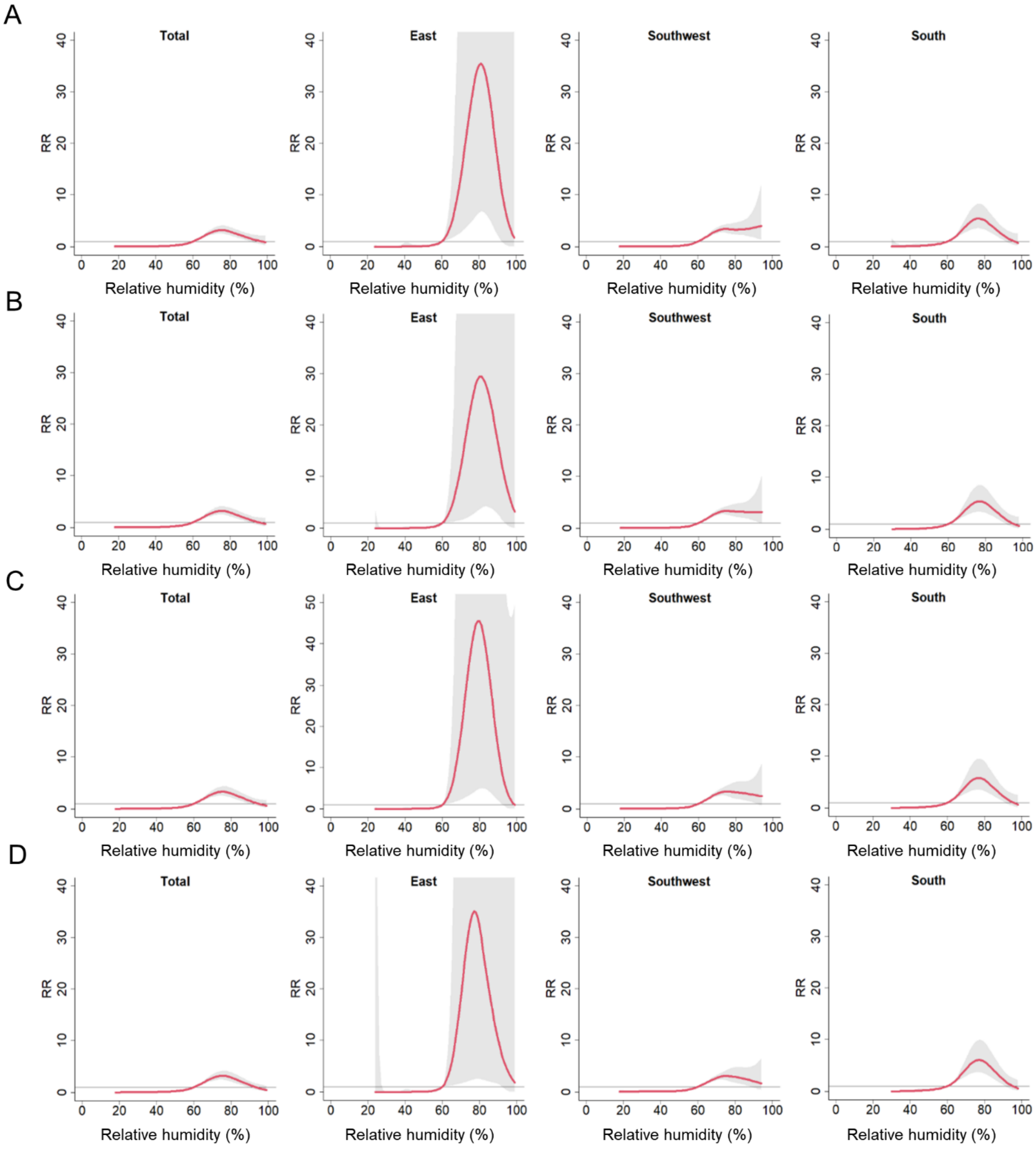

Supplementary Figure 3. Sensitivity analysis of associations between weekly relative humidity and scrub typhus incidence by altering the maximum lag time period to (A) 14, (B) 16, (C) 18 and (D) 20 weeks, respectively.

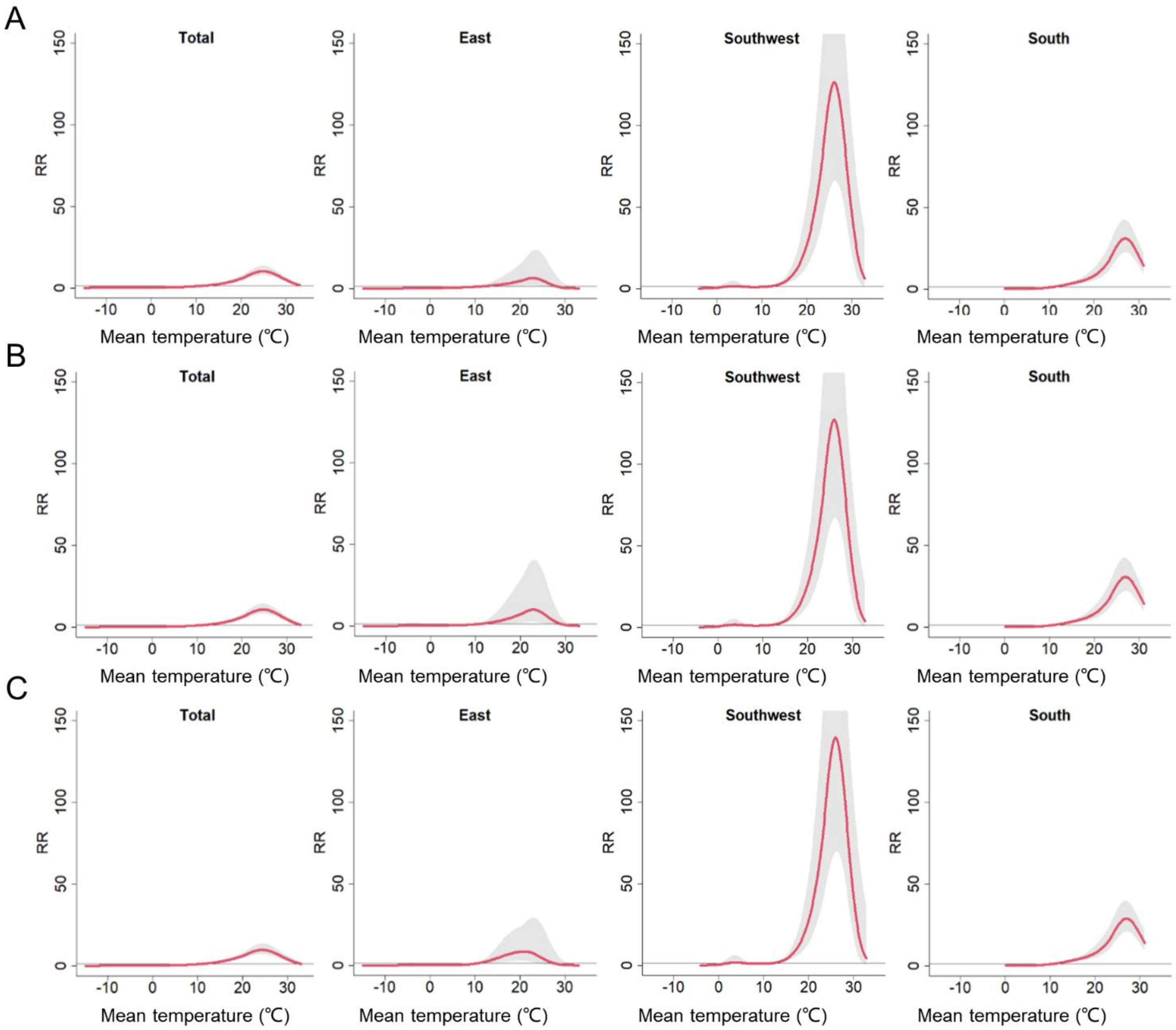

Supplementary Figure 4. Sensitivity analysis of associations between weekly mean temperature and scrub typhus incidence by altering the degree freedom values of time to (A) 6, (B) 8 and (C) 10, respectively.

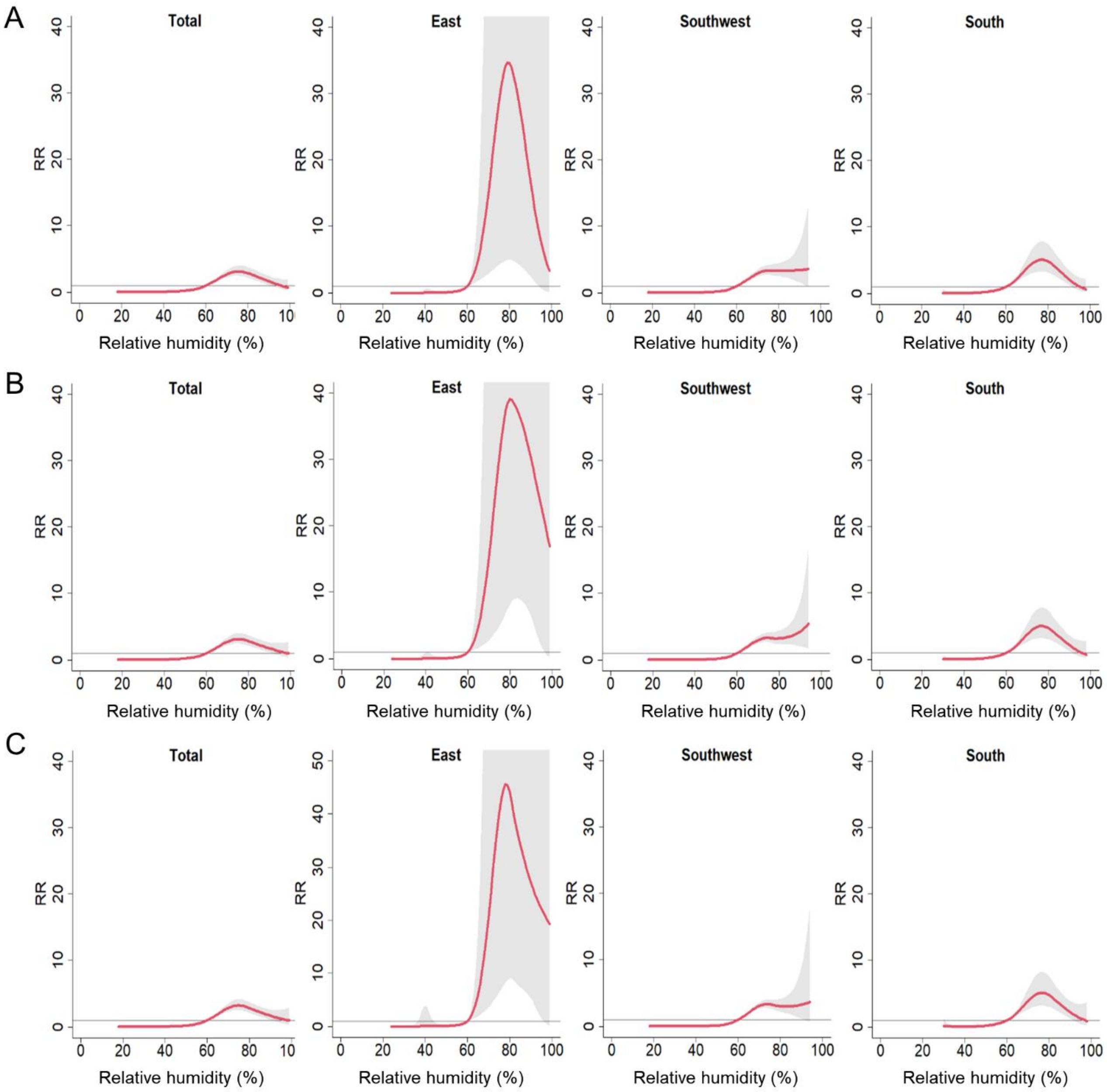

Supplementary Figure 5. Sensitivity analysis of associations between weekly relative humidity and scrub typhus incidence by altering the degree freedom values of time to (a) 6, (b) 8 and (c)10, respectively.

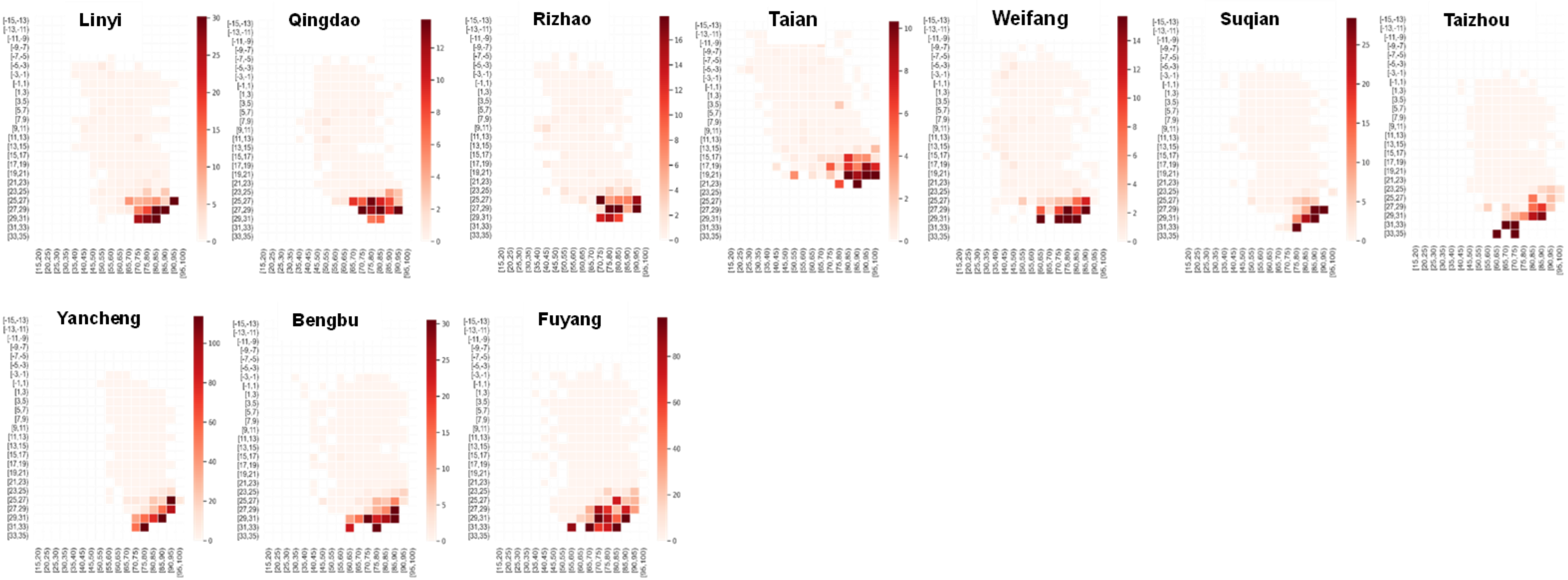

Supplementary Figure 6. Heatmaps of scrub typhus cases against mean temperature and relative humidity in the 11 prefecture-level administrative regions of the East. The horizontal axis denotes mean temperature intervals and the vertical axis denotes relative humidity intervals. Each cells denotes the mean scrub typhus cases under the corresponding climate conditions.

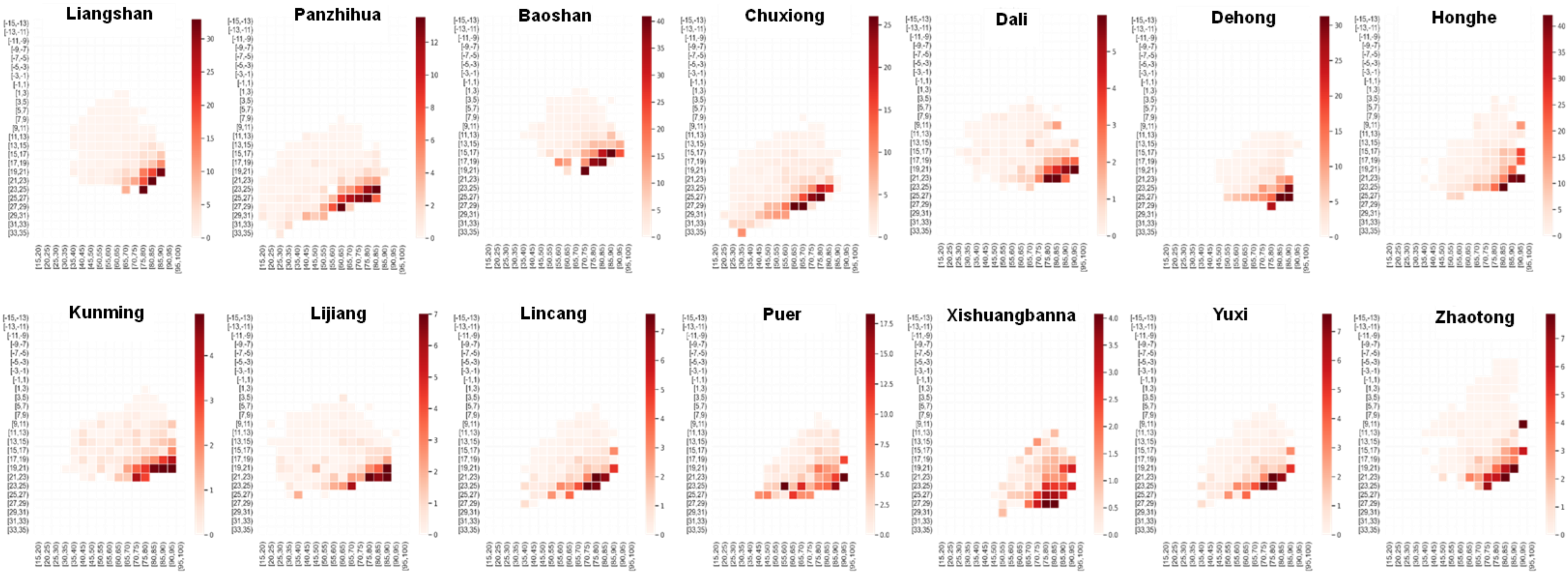

Supplementary Figure 7. Heatmaps of scrub typhus cases against mean temperature and relative humidity in the 14 prefecture-level administrative regions of the Southwest. The horizontal axis denotes mean temperature intervals and the vertical axis denotes relative humidity intervals. Each cells denotes the mean scrub typhus cases under the corresponding climate conditions.

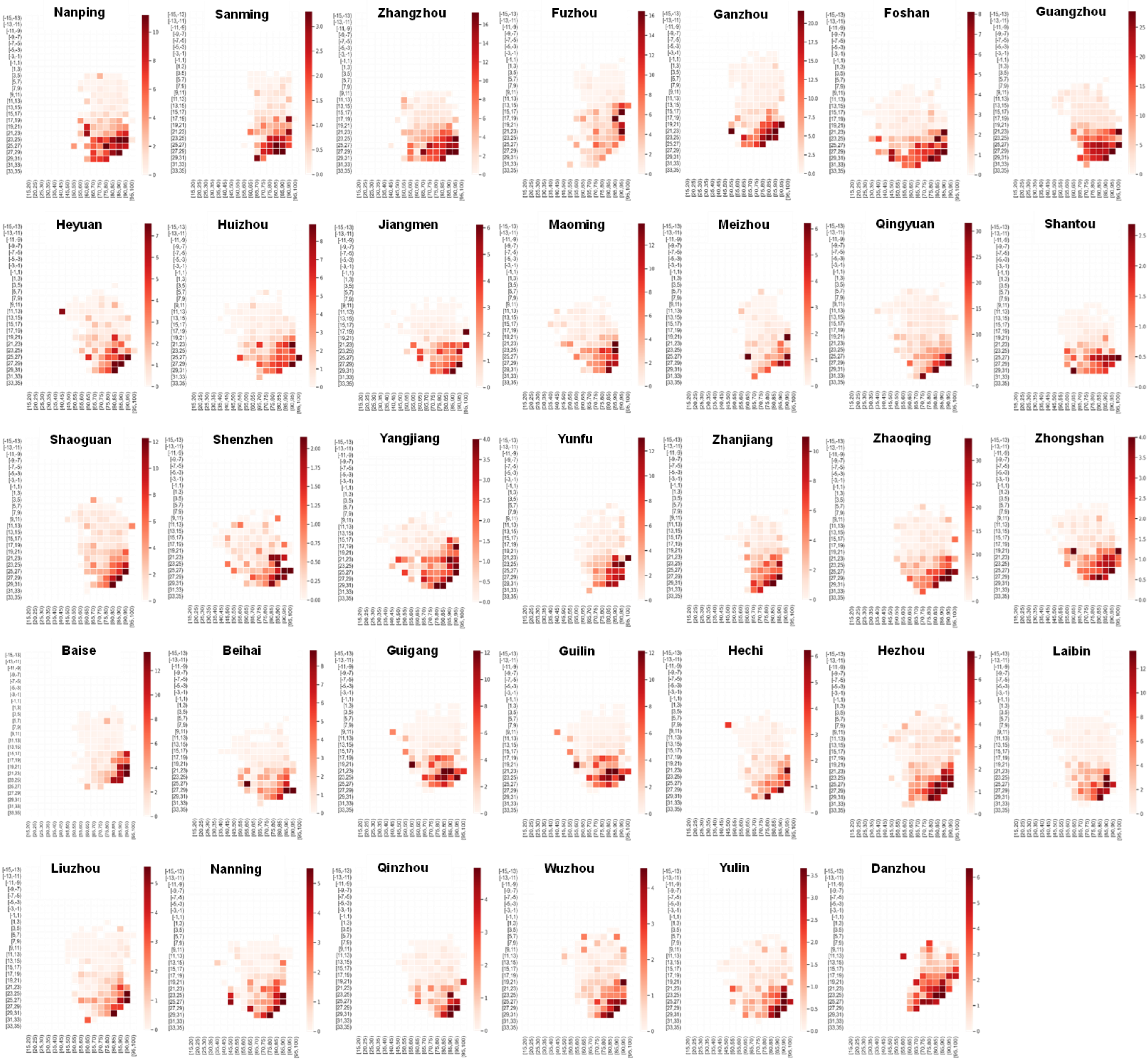

Supplementary Figure 8. Heatmaps of scrub typhus cases against mean temperature and relative humidity in the 34 prefecture-level administrative regions of the South. The horizontal axis denotes mean temperature intervals and the vertical axis denotes relative humidity intervals. Each cells denotes the mean scrub typhus cases under the corresponding climate conditions.

1.2 Supplementary Tables

Supplementary Table 1. The number of reported scrub typhus cases and the annual meteorological conditions in the 59 prefecture-level administrative regions during 2006 to 2020.

Location	Province	Region	Scrub typhus cases		Meteorological factors				
			Total number	Age (years)	MT (℃)	RH (%)	Rainfall (mm)	SSH (h)	Wind velocity (m/s)
Linyi	Shandong	East	2536	59	14.2	67.4	2.2	6	1.9
Qingdao	Shandong	East	1054	59	13.3	68.7	1.9	6.3	3
Rizhao	Shandong	East	1208	58	13.7	68.4	2.2	5.8	2.2
Taian	Shandong	East	1156	56	6.5	64.6	2.9	7	6.4
Weifang	Shandong	East	1092	57	13.8	63.9	1.7	6.4	2.1
Suqian	Jiangsu	East	1738	60	15.3	72.6	2.7	5.3	2
Taizhou	Jiangsu	East	2293	63	16.6	73.3	3.4	5.3	2
Yancheng	Jiangsu	East	5296	62	15.2	76.6	2.9	5.6	2.5
Bengbu	Anhui	East	2173	60	15.9	72.5	2.9	5.4	2.4
Bozhou	Anhui	East	3432	56	15.8	69.4	2.4	5.4	2.2
Fuyang	Anhui	East	7357	58	15.8	73.4	2.6	4.8	2.4
Liangshan	Sichuan	Southwest	3084	37	14.4	65.7	2.7	5.6	1.6
Panzhihua	Sichuan	Southwest	2233	42	21.2	54.9	2.2	7.5	1.5
Baoshan	Yunnan	Southwest	10727	41	16.2	73.4	3.6	6.6	1.8
Chuxiong	Yunnan	Southwest	4464	48	22	56.8	1.7	6.8	2
Dali	Yunnan	Southwest	958	51	15.7	65.6	2.9	6	2.2
Dehong	Yunnan	Southwest	6674	38	21.1	75.2	3.8	6.3	1.1
Honghe	Yunnan	Southwest	5440	39	18	74.7	2.9	5.4	2.2
Kunming	Yunnan	Southwest	1014	45	15.1	71.5	2.7	6.1	2.8
Lijiang	Yunnan	Southwest	799	48	15.8	60	2.7	6.7	2.4
Lincang	Yunnan	Southwest	9699	38	19.5	71.4	3.1	6.4	1.1
Puer	Yunnan	Southwest	3867	45	19.6	76.4	4.4	5.7	1
Xishuangbanna	Yunnan	Southwest	1470	34	22.8	77.3	3.5	6	1
Yuxi	Yunnan	Southwest	1658	51	20.7	67.5	2.3	6.2	2.1
Zhaotong	Yunnan	Southwest	919	43	12.5	73.4	2.2	4.8	1.7
Nanping	Fujian	South	2871	55	19.1	78.3	5.1	4.5	1.3
Sanming	Fujian	South	1011	56	19.2	79.7	4.8	4.4	1.3

Zhangzhou	Fujian	South	5009	53	22.1	75	4	5.5	3.3
Fuzhou	Jiangxi	South	2133	52	18.6	78.6	5.1	4.4	1.8
Ganzhou	Jiangxi	South	5130	56	19.6	75.9	4.6	4.5	1.6
Foshan	Guangdong	South	2255	57	23.7	71.8	5.6	4.7	2.2
Guangzhou	Guangdong	South	9481	55	22.2	79.6	6.1	4.6	1.9
Heyuan	Guangdong	South	1828	58	21.3	75.8	4.9	4.6	1.7
Huizhou	Guangdong	South	2122	54	22.6	75.1	5.4	4.5	2
Jiangmen	Guangdong	South	1636	58	23.1	79.7	6.1	5.1	3.3
Maoming	Guangdong	South	3833	58	23.3	79.1	5	5.1	2.3
Meizhou	Guangdong	South	922	59	21.9	76.5	4.5	4.9	1.7
Qingyuan	Guangdong	South	5994	55	21.3	76.2	5.5	4.5	2
Shantou	Guangdong	South	644	56	22.7	76.6	4.1	5.5	2.2
Shaoguan	Guangdong	South	2766	54	20.5	77.5	4.9	4.4	2
Shenzhen	Guangdong	South	545	47	23.4	74	5.5	5.1	2.1
Yangjiang	Guangdong	South	1288	60	22.8	80.4	6.9	4.7	3.7
Yunfu	Guangdong	South	3413	56	22.7	79.6	4.1	4.3	1.6
Zhanjiang	Guangdong	South	3322	59	23.9	82.8	4.7	5.4	2.9
Zhaoqing	Guangdong	South	8969	57	22.2	77.7	4.9	4.2	1.6
Zhongshan	Guangdong	South	1208	53	23	76.6	5.6	5	1.9
Baise	Guangxi	South	1843	50	21.5	76.3	3.7	3.9	1.5
Beihai	Guangxi	South	1676	57	23.4	80.4	5	5.2	3.1
Guigang	Guangxi	South	3484	59	22.2	78.4	5.2	4	1.5
Guilin	Guangxi	South	709	56	19.7	74.4	5.5	3.7	1.8
Hechi	Guangxi	South	1097	54	20.8	77.4	4.5	3.7	1.7
Hezhou	Guangxi	South	2000	54	20.5	75.8	4.9	4	1.9
Laibin	Guangxi	South	2965	56	21.5	75.7	4.2	4	1.5
Liuzhou	Guangxi	South	782	55	20.4	75	5	3.8	1.8
Nanning	Guangxi	South	1275	60	21.8	78.6	3.8	4.2	1.8
Qinzhou	Guangxi	South	1151	58	22.6	79.1	5.6	4.5	2.2
Wuzhou	Guangxi	South	1168	56	20.9	78.3	5	4.2	1.9
Yulin	Guangxi	South	846	55	22.6	77.1	4.7	4.2	1.8
Danzhou	Hainan	South	2865	51	24.4	80.4	6.2	5.3	1.6

Note 1: Age is denoted as median. MT is the abbreviation for mean temperature; RH is the abbreviation for relative humidity; SSH is the abbreviation for sunshine hours.

Supplementary Table 2. Lag weeks with the maximum Spearman correlation coefficients of scrub typhus cases with mean temperature and relative humidity.

Location	Region	Lag week	Location	Region	Lag week
Weifang	East	12	Guilin	South	4
Qingdao	East	12	Liuzhou	South	5
Rizhao	East	12	Hechi	South	2
Taian	East	12	Hezhou	South	4
Linyi	East	12	Laibin	South	3
Bozhou	East	12	Baise	South	2
Suqian	East	13	Wuzhou	South	5
Yancheng	East	13	Guigang	South	3
Bengbu	East	13	Nanning	South	6
Taizhou	East	14	Yulin	South	5
Fuyang	East	13	Qinzhou	South	2
Liangshan	Southwest	2	Beihai	South	5
Panzhihua	Southwest	2	Shaoguan	South	5
Zhaotong	Southwest	2	Meizhou	South	4
Lijiang	Southwest	4	Shantou	South	2
Dali	Southwest	2	Heyuan	South	5
Chuxiong	Southwest	5	Qingyuan	South	3
Kunming	Southwest	3	Zhaoqing	South	4
Baoshan	Southwest	4	Guangzhou	South	3
Yuxi	Southwest	3	Foshan	South	3
Dehong	Southwest	4	Yunfu	South	5
Lincang	Southwest	4	Huizhou	South	6
Puer	Southwest	5	Shenzhen	South	6
Honghe	Southwest	1	Zhongshan	South	3
Xishuangbanna	Southwest	2	Jiangmen	South	4
Nanping	South	2	Yangjiang	South	7
Sanming	South	2	Maoming	South	3
Zhangzhou	South	5	Zhanjiang	South	6
Fuzhou	South	3	Danzhou	South	3
Ganzhou	South	4			
